# Vimentin Is an Attachment Receptor for Mycoplasma pneumoniae P1 Protein

**DOI:** 10.1128/spectrum.04489-22

**Published:** 2023-03-13

**Authors:** Kailan Peng, Yating Liao, Xia Li, Dongdong Zeng, Youyuan Ye, Li Chen, Zhuo Zeng, Yanhua Zeng

**Affiliations:** a Institute of Pathogenic Biology, Basic Medical School, Hengyang Medical College, Hunan Provincial Key Laboratory for Special Pathogens Prevention and Control, University of South China, Hengyang, Hunan Province, People’s Republic of China; b Department of Cardiocascular Medicine, the Third Affiliated Hospital, University of South China, Hengyang, Hunan Province, People’s Republic of China; Griffith University

**Keywords:** *Mycoplasma pneumoniae*, P1 protein, vimentin, BEAS-2B, receptor

## Abstract

Mycoplasma pneumoniae is the most common pathogen causing respiratory tract infection, and the P1 protein on its adhesion organelle plays a crucial role during the pathogenic process. Currently, there are many studies on P1 and receptors on host cells, but the adhesion mechanism of P1 protein is still unclear. In this study, a modified virus overlay protein binding assay (VOPBA) and liquid chromatography-mass spectrometry (LC-MS) were performed to screen for proteins that specifically bind to the region near the carboxyl terminus of the recombinant P1 protein (rP1-C). The interaction between rP1-C and vimentin or β-4-tubulin were confirmed by far-Western blotting and coimmunoprecipitation. Results verified that vimentin and β-4-tubulin were mainly distributed on the cell membrane and cytoplasm of human bronchial epithelial (BEAS-2B) cells, but only vimentin could interact with rP1-C. The results of the adhesion and adhesion inhibition assays indicated that the adhesion of M. pneumoniae and rP1-C to cells could be partly inhibited by vimentin and its antibody. When vimentin was downregulated with the corresponding small interfering RNA (siRNA) or overexpressed in BEAS-2B cells, the adhesion of M. pneumoniae and rP1-C to cells was decreased or increased, respectively, which indicated that vimentin was closely associated with the adhesion of M. pneumoniae and rP1-C to BEAS-2B cells. Our results demonstrate that vimentin could be a receptor on human bronchial epithelial cells for the P1 protein and plays an essential role in the adhesion of M. pneumoniae to cells, which may clarify the pathogenesis of M. pneumoniae.

**IMPORTANCE** Mycoplasma pneumoniae is the most common pathogen causing respiratory tract infection, and the P1 protein on its adhesion organelle plays a crucial role during the pathogenic process. A variety of experiments, including enzyme-linked immunosorbent assay (ELISA), coimmunoprecipitation, adhesion, and adhesion inhibition assay, have demonstrated that the M. pneumoniae P1 protein can interact with vimentin, that the adhesion of M. pneumoniae and recombinant P1 protein to BEAS-2B cells was affected by the expression level of vimentin. This provides a new idea for the prevention and treatment of Mycoplasma pneumoniae infection.

## INTRODUCTION

Mycoplasma pneumoniae is a wall-less bacterium that causes respiratory tract infections such as pneumonia, pharyngitis, and tracheitis (1). As one of the leading causes of community-acquired pneumonia (CAP), M. pneumoniae infects only humans and is transmitted through droplets and aerosols (2). Infections often erupt in schools, the military, communities, and other crowded places (3), with clinical manifestations of cough, fever, headache, muscle soreness, and general fatigue (4). In addition, M. pneumoniae infection can cause extrapulmonary complications, including central nervous system disorders such as encephalitis (5) and optic neuritis (6), and skin ailments such as maculopapules and herpes zoster (7).

The pathogenic mechanism of M. pneumoniae includes adhesion, toxin release, and host inflammatory responses (8, 9). The adhesion of M. pneumoniae to respiratory epithelial cells is the initial step in its pathogenesis, which is mediated by the attachment organelle. Previous studies demonstrated that strains lacking adhesion capacity also generally lack pathogenicity (10). Currently, studies have shown that the attachment organelle is mainly composed of P1, P30, P40, P90, and high molecular weight proteins 1 (HMW1) and 3 (HMW3). The P1 protein, with a molecular weight of 170 kDa, is one of the most immunodominant proteins mediating the adhesion of M. pneumoniae to the host cell membrane and can directly bind to the membrane surface receptors. Widjaja et al. showed that the C-terminal region of P1 can selectively bind to cytokeratin and vimentin (11). Aggregation of P1 at the tip of the apical organelle is a decisive factor for successful adhesion, and both antisera against P1 and mutants lacking P1 prevent adhesion and gliding motility of *Mycoplasma* (12, 13). Prior studies determined that the adhesion epitopes of P1 protein were located at its carboxyl terminus (14, 15), which has strong immunogenicity (16). In addition to directly inducing lung inflammation, P1 protein may also cause an autoimmune response or act as an immune decoy after being released into the extracellular milieu (11). There is no doubt that P1 protein plays an essential role in the pathogenesis of M. pneumoniae.

Receptors on host cells, especially those interacting directly with pathogens, are essential for microbial pathogenesis. Colonization by nontypeable Haemophilus influenzae and its survival in host serum are determined by protein E interactions with laminin and vitronectin (17). Studies also validated that Staphylococcus aureus, Streptococcus pyogenes, Enterococcus faecium, etc., cause disease by binding to fibronectin (18). Therefore, receptors on host cells are inextricably linked to colonization by pathogens. In addition, changes in signal transduction after the pathogen binds to the receptor also promote further invasion, pathogenesis, and proliferation of the microbes. The VP4 spike protein of rotavirus enhances viral assembly by interacting with the host prenylated Rab acceptor domain family member 1 (PRAF1) (19). The release of infectious yellow fever virus particles was mediated by the interaction between the yellow fever virus nonstructural 3 (NS3) protein and the apoptosis-linked gene 2-interacting protein X (ALIX) (20). Thus, investigating the interactions between pathogens and receptors can provide a better understanding of the mechanisms exploited by bacteria, which may help to identify new potential drug targets.

In this research, we expressed and purified amino acid residues 1160 to 1498 at the carboxyl terminus of P1 protein, screened human BEAS-2B bronchial epithelial cells for corresponding interacting proteins from membranes by using a modified virus overlay protein binding assay (VOPBA), and found that the recombinant the domain near the C terminus of P1 protein (rP1-C) could interact with vimentin. The adhesion of rP1-C and M. pneumoniae to BEAS-2B cells was affected by changes in vimentin expression. Therefore, we concluded that vimentin might serve as a receptor for the adhesion of M. pneumoniae to host cells via P1 protein.

## RESULTS

### Expression, purification, and identification of rP1-C.

**FIG 1:**
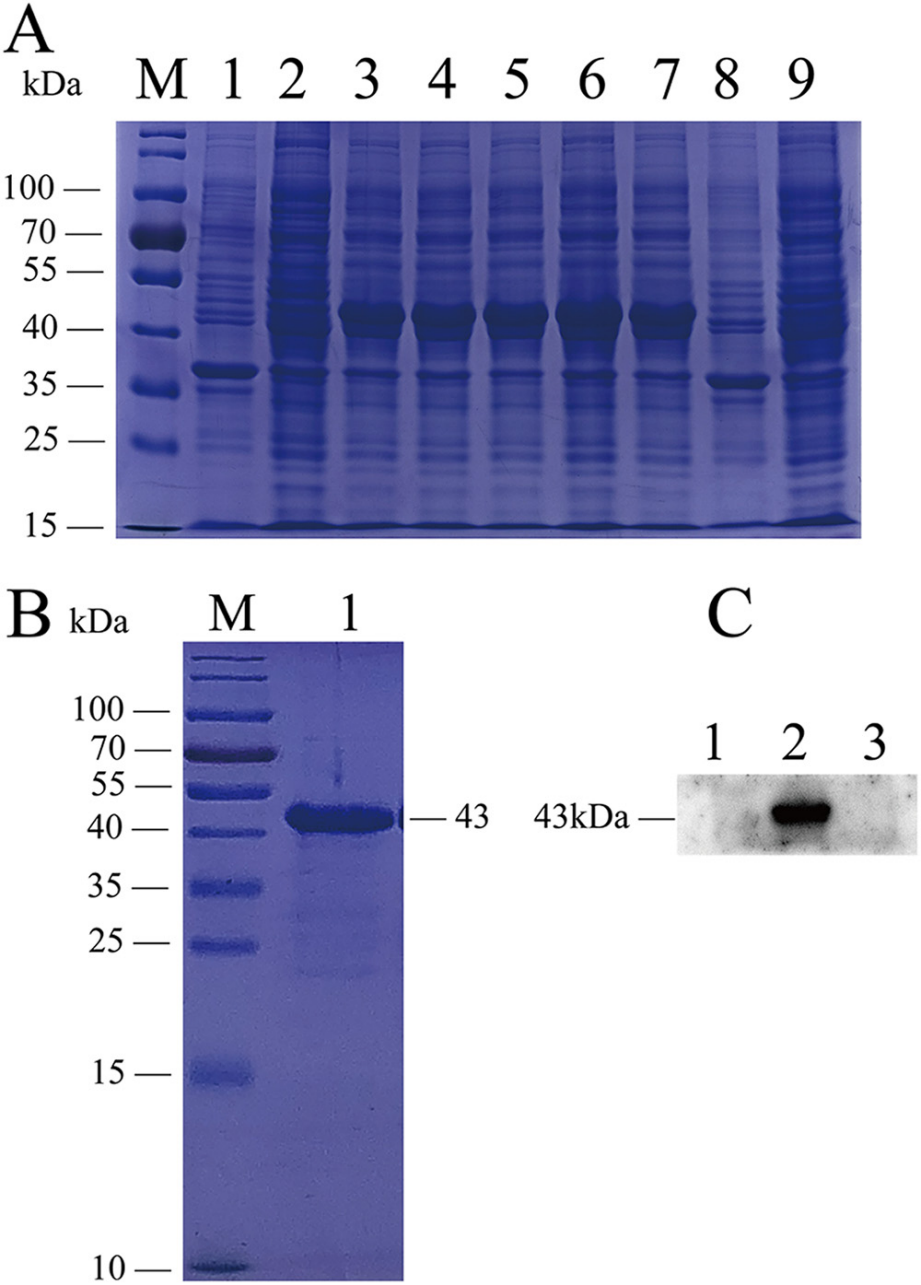
Expression, purification, and identification of recombinant His-tagged P1 protein. (A) SDS-PAGE analysis of protein expression. Lanes 1 and 8, supernatants of E. coli without IPTG; lanes 2 and 9, precipitation of E. coli without IPTG; lanes 3 to 7, supernatants of E. coli added with different concentrations of IPTG. (B) SDS-PAGE analysis of purified, concentrated rP1-C. (C) Verification of protein expression by Western blotting using anti-His antibody. Lane 1, empty E. coli suspension; lane 2, IPTG group; lane 3, no-IPTG group. M, molecular weight markers.

The fragment of the M. pneumoniae *p1* gene was cloned into the expression vector pET-30a(+) and expressed in Escherichia coli cells. The expressed proteins were analyzed by SDS-PAGE and Western blotting. As shown in Fig. 1A, there was a prominent band at a molecular weight of about 43 kDa, indicating that the protein was successfully expressed. The expressed and purified proteins were analyzed by SDS-PAGE. The staining results showed a single band at 43 kDa (Fig. 1B). The purified protein was further confirmed by Western blotting using an anti-His antibody (Fig. 1C), and the concentration was determined to be about 1.5 mg/mL.

### Preparation and purification of rP1-C polyclonal antibody.

To prepare polyclonal antibody against rP1-C, rabbits were immunized with rP1-C. The serum was collected, precipitated with saturated ammonium sulfate, and purified using rP1-C-conjugated CNBr-activated Sepharose 4B. As shown in Fig. S2A in the supplemental material, the molecular weights of the heavy chain and light chain of the polyclonal antibody were about 50 kDa and 25 kDa, respectively. The specificity of the purified serum was determined by Western blotting using rP1-C as the antigen. There was a band at a molecular weight of about 43 kDa, which demonstrated that the rabbit-derived rP1-C polyclonal antibody was successfully prepared and purified (Fig. S2B).

### A 55-kDa cell membrane protein binds to rP1-C.

**FIG 2:**
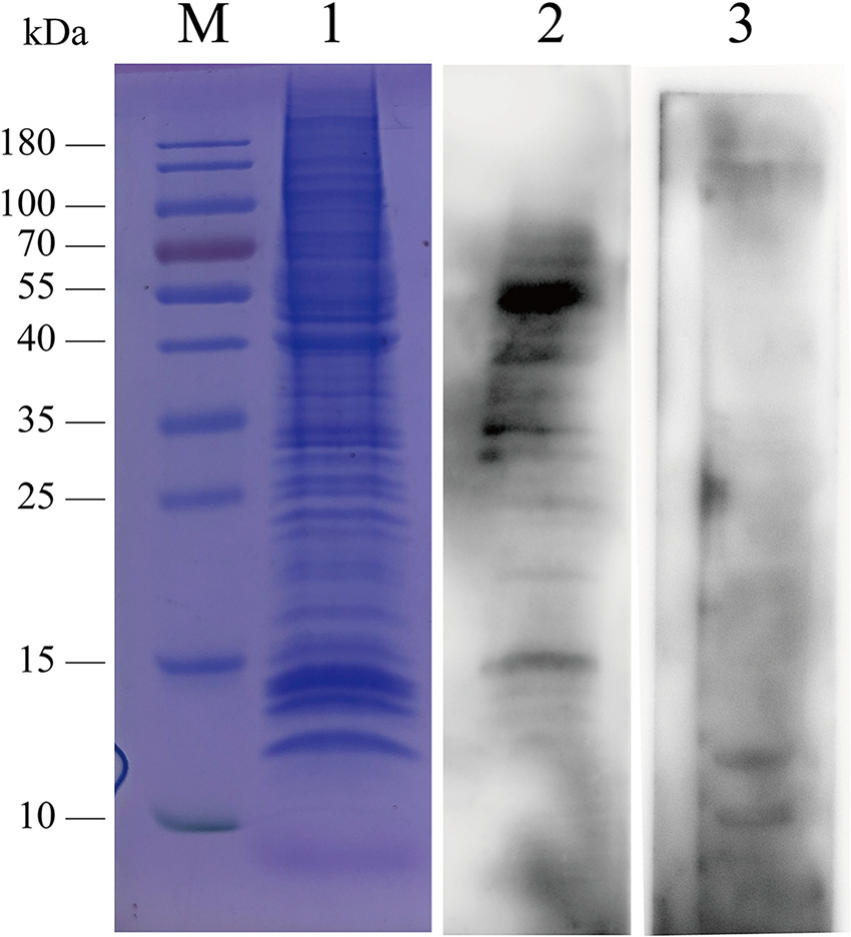
Screening of BEAS-2B membrane fraction for proteins that specifically bind to rP1-C. Lane 1, SDS-PAGE analysis of extracted BEAS-2B cell membrane proteins; lane 2, extracted membrane protein transferred to PVDF membranes and preincubated with rP1-C; lane 3, extracted membrane protein transferred to PVDF membranes and preincubated with BSA.

BEAS-2B cell membrane proteins were extracted (1.1 mg/mL) and subjected to SDS-PAGE. The results demonstrated that the molecular weights of membrane proteins were mainly distributed over a range of 10 to 200 kDa (Fig. 2). To identify the specific binding protein of rP1-C, a modified VOPBA was performed. As shown in Fig. 2, there were some distinct bands on the gel, among which the one at 55 kDa was the strongest, while no band was observed at the corresponding position for the control group. These results demonstrated that there were one or more BEAS-2B cell membrane proteins that bound to rP1-C, and the most prominent one had a molecular weight of 55 kDa.

### Vimentin and β-4-tubulin are the main components of the 55-kDa membrane protein.

To identify the main protein component that specifically bound explicitly to rP1-C, the band at 55 kDa was cut out for liquid chromatography-mass spectrometry (LC-MS) analysis. According to the protein matching and comparison results from the NCBI database retrieval, it was found that vimentin and β-4-tubulin had the highest scores (Table 1). Therefore, vimentin and β-4-tubulin might be the main components of the 55-kDa membrane protein band, responsible for the specific binding of the rP1-C protein.

**TABLE 1:** Protein components at approximately 55 kDa

Accession	Score	Mass	No. of matches	No. of sequences	emPAI^*a*^	Protein description^*b*^
VIME_HUMAN	1895	53,676	72	29	11.91	Vimentin OS=Homo sapiens OX=9606 GN=VIM PE=1 SV=4
TBB4B_HUMAN	1369	50,225	84	18	8.23	Tubulin beta-4B chain OS=Homo sapiens OX=9606 GN=TUBB4B PE=1 SV=1

a emPAI, exponentially modified protein abundance index.

b The item in the last column is a description of the protein. Vimentin and Tubulin β-4B chain are the protein names. OS, organism; GN, gene name, PE, protein existence (representing protein reliability), and SV, sequence version, these are uniprot database entries.

**FIG 3:**
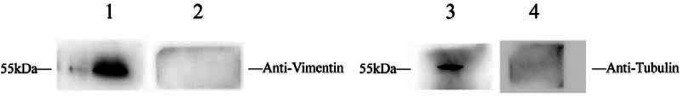
Confirming the existence of vimentin in BEAS-2B cell membrane protein. Lane 1, extracted membrane protein incubated with anti-vimentin antibody; lane 2, extracted membrane protein incubated with IgG; lane 3, extracted membrane protein incubated with anti-β-4-tubulin antibody; lane 4, extracted membrane protein incubated with IgG.

Western blotting was used to confirm whether the extracted membrane proteins contained vimentin and β-4-tubulin. A clear band at a molecular weight of about 55 kDa was observed in the experimental group incubated with an anti-vimentin antibody or anti-β-4-tubulin antibody, whereas no band was observed in the control group (Fig. 3). These results indicated that the membrane proteins at 55 kDa did contain vimentin and β-4-tubulin.

### Vimentin and β-4-tubulin are distributed on the BEAS-2B cell membrane and in the cytoplasm.

**FIG 4:**
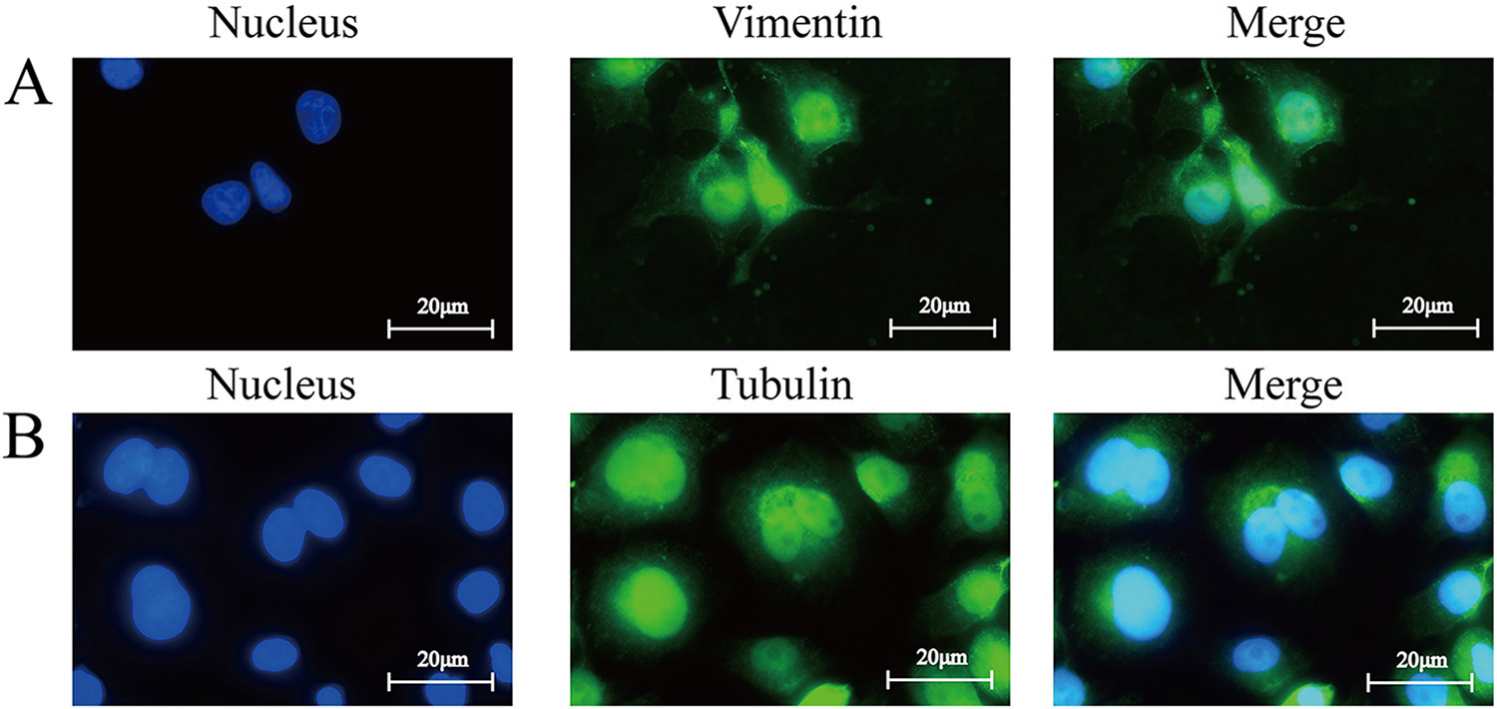
Distribution of vimentin and β-4-tubulin in BEAS-2B cells. The cells were fixed and blocked, the nucleus was stained with DAPI (blue), and the vimentin and β-4-tubulin on the cells were stained with FITC (green). All signals were merged (×1,000, in oil, inverted microscope; Nikon).

An indirect immunofluorescence assay was performed to determine the distribution of vimentin and β-4-tubulin in the BEAS-2B cell line (Fig. 4). Vimentin and β-4-tubulin (green fluorescence) were widely distributed on the membrane and cytoplasm of BEAS-2B cells, supporting the idea that vimentin and β-4-tubulin are mainly located on the surface and in the cytoplasm of BEAS-2B cells.

### rP1-C and M. pneumoniae colocalized with vimentin or β-4-tubulin on the membrane surface of BEAS-2B cells.

**FIG 5:**
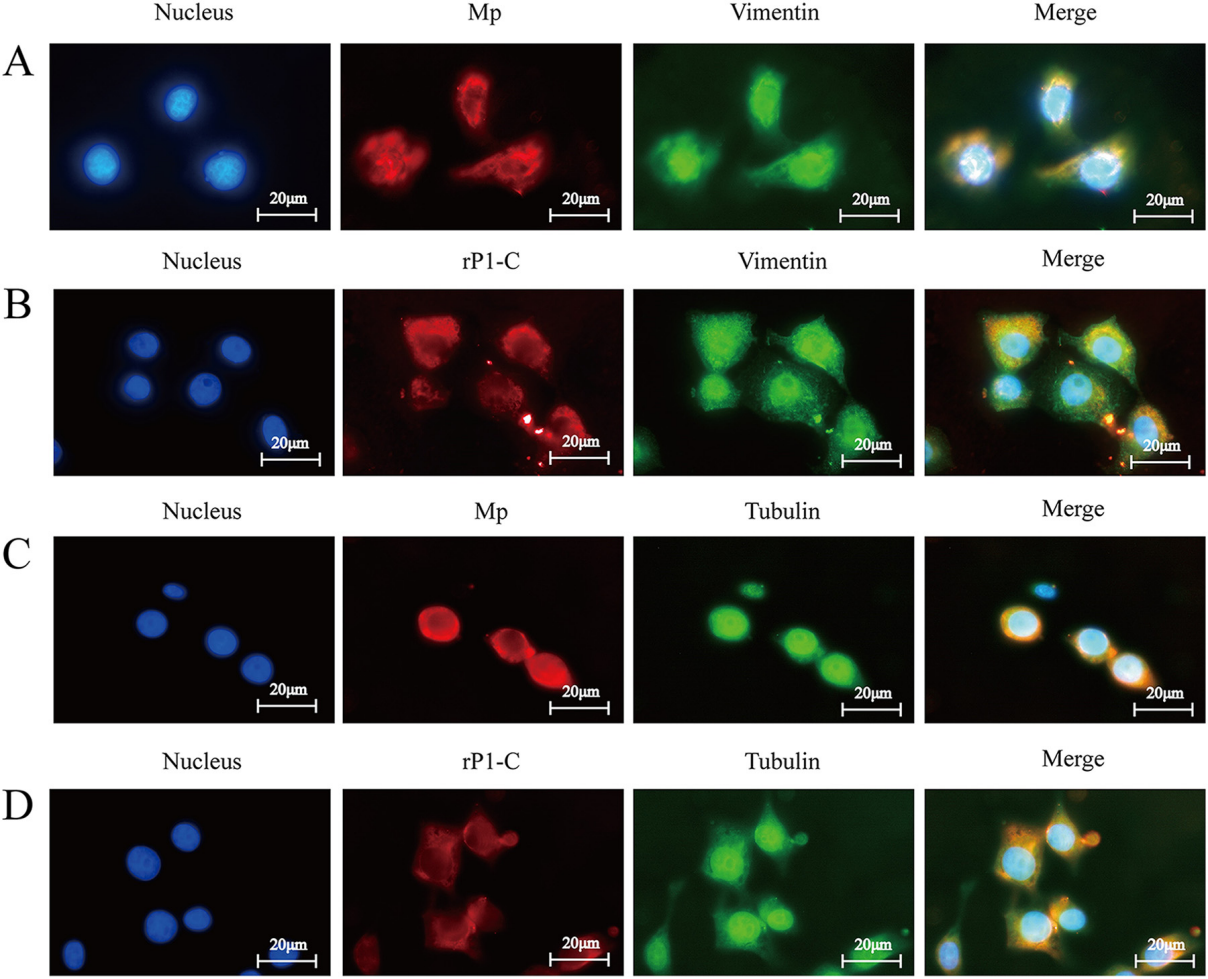
Colocalization of vimentin with M. pneumoniae and rP1-C. (A) Colocalization of vimentin with M. pneumoniae. (B) Colocalization of vimentin with rP1-C. (C) Colocalization of β-4-tubulin with M. pneumoniae. (D) Colocalization of β-4-tubulin with rP1-C. Vimentin and β-4-tubulin were stained into green, while M. pneumoniae and rP1-C were stained into red. All signals were merged (yellow) (×1000, in oil, inverted microscope; Nikon).

Colocalization analyses were executed to determine whether rP1-C and M. pneumoniae could bind to vimentin or β-4-tubulin on BEAS-2B cells (Fig. 5). BEAS-2B cells were preincubated with rP1-C protein fragment or M. pneumoniae and stained with a combination of the rP1-C antibodies or M. pneumoniae antibody and vimentin or β-4-tubulin antibody. Representative confocal images in Fig. 5 reveal that the rP1-C protein fragment and M. pneumoniae (red fluorescence) were mainly distributed on the membrane of BEAS-2B cells, overlapping with vimentin or β-4-tubulin (green fluorescence). The merged images (yellow) illustrate that rP1-C and M. pneumoniae may interact with vimentin or tubulin on the cell surface.

### Vimentin interacts with rP1-C.

**FIG 6:**
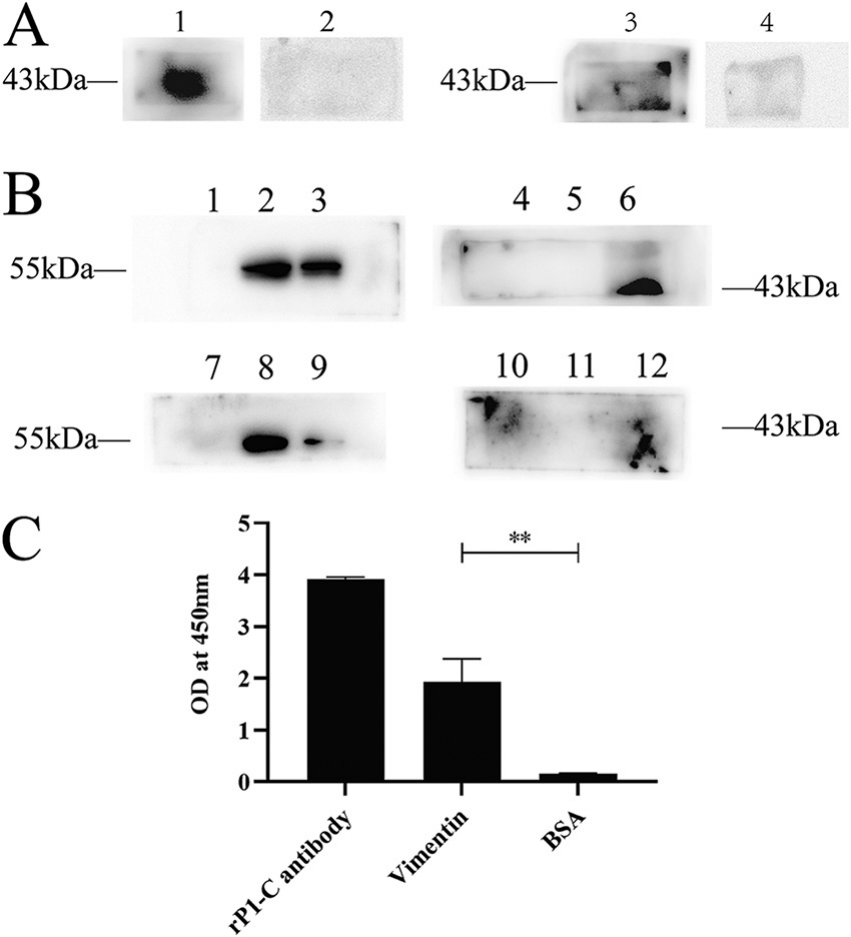
Vimentin can bind to rP1-C *in vitro*. (A) Far-Western blotting of rP1-C. Lane 1, purified rP1-C, preincubated with cell membrane proteins and incubated with anti-vimentin antibody; lane 2, purified rP1-C, preincubated with BSA and incubated with anti-vimentin antibody; lane 3, purified rP1-C, preincubated with cell membrane proteins and incubated with anti-tubulin antibody; lane 4, purified rP1-C, preincubated with BSA and incubated with anti-β-4-tubulin antibody. (B) Lane 1, sample precipitated by IgG; lane 2, input; lane 3, rP1-C–vimentin complex precipitated by anti-rP1-C antibody, incubated with anti-vimentin antibody, and visualized; lane 4, sample precipitated by IgG; lane 5, input; lane 6, vimentin–rP1-C complex precipitated by anti-vimentin antibody, incubated with anti-rP1-C antibody, and visualized; lane 7, sample precipitated by IgG; lane 8, input; lane 9, rP1-C–β-4-tubulin complex precipitated by anti-rP1-C antibody, incubated with anti-tubulin antibody, and visualized; lane 10, sample precipitated by IgG; lane 11, input; lane 12, β-4-tubulin–rP1-C complex precipitated by anti-tubulin antibody, incubated with anti-rP1-C antibody, and visualized; lysate of BEAS-2B cell was taken as input. (C) Indirect ELISA between rP1-C and vimentin. rP1-C was incubated with vimentin, anti-rP1-C antibody, or BSA. rP1-C was used as a positive control and BSA was used as a negative control.

To determine if vimentin and β-4-tubulin could interact with rP1-C, far-Western blotting, coimmunoprecipitation assays, and indirect enzyme-linked immunosorbent assay (ELISA) were performed. For the far-Western blotting, the vimentin group, the membrane protein preincubation group, showed a distinct band at 43 kDa (Fig. 6A). In contrast, the control bovine serum albumin (BSA) preincubation group showed no band, indicating that vimentin could interact with rP1-C. There were no apparent bands in the tubulin group, indicating that β-4-tubulin did not appear to bind to rP1-C or there was weak binding between the two. In the coimmunoprecipitation assay, prominent bands were observed in lane 3 and lane 6. In contrast, no specific bands were observed for the corresponding IgG groups (Fig. 6B). The distinct immunoblot bands demonstrated that the complex formed by rP1-C and vimentin could be precipitated by rP1-C antibody or vimentin antibody. However, the coimmunoprecipitation of tubulin and rP1-C was not efficient. Therefore, we did not conduct a follow-up experiment on tubulin. The indirect ELISA results revealed that the absorbance of the experimental group was significantly higher than that of the negative-control group, which meant that vimentin showed a high degree of specificity for rP1-C (Fig. 6C). Taken together, these results confirmed that vimentin could specifically interact with rP1-C.

### Adhesion and adhesion inhibition assays.

**FIG 7:**
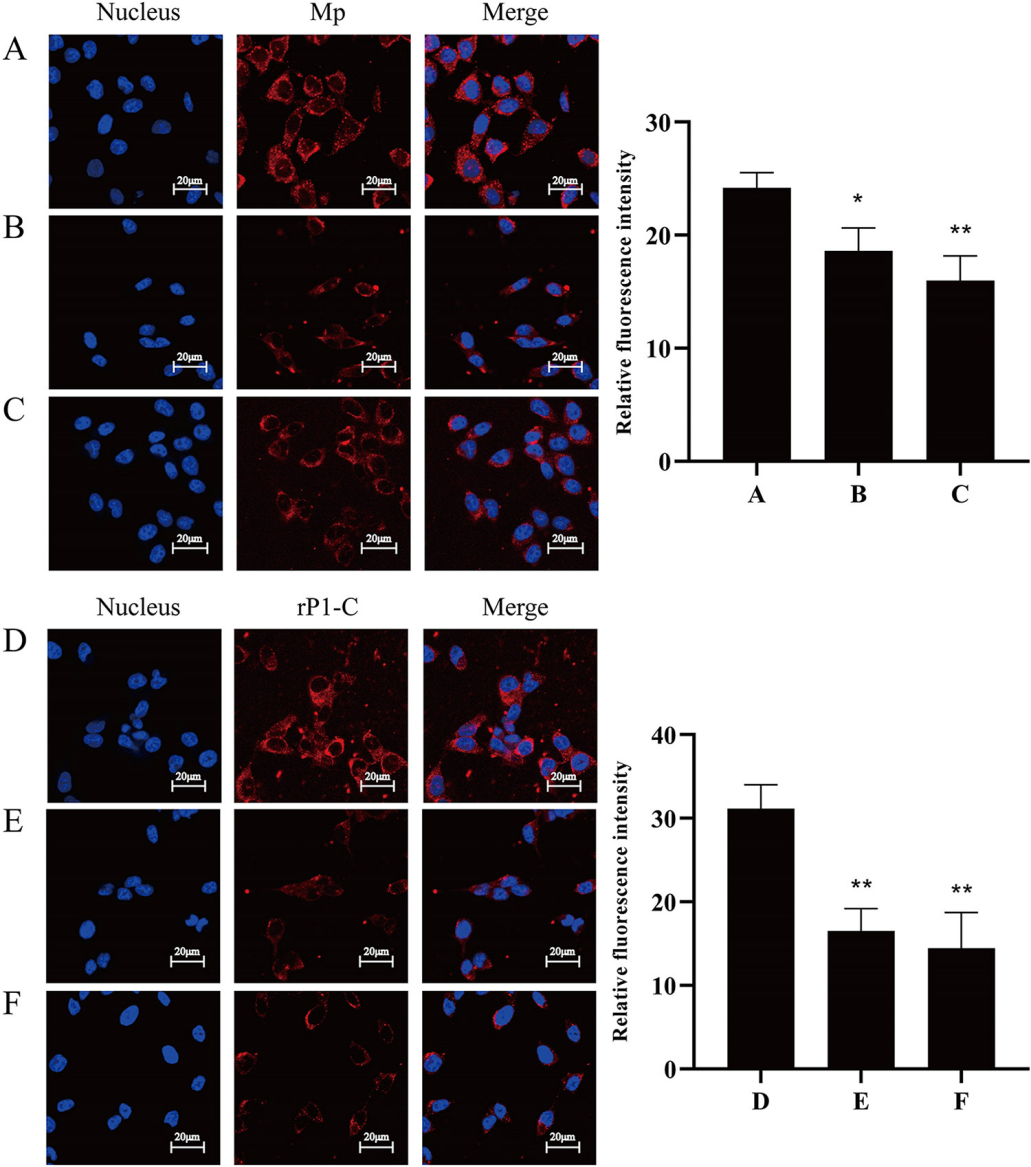
Adhesion and adhesion inhibition assay. (A and D) Control group incubated with M. pneumoniae directly; (B and E) anti-vimentin antibody-preincubated group; (C and F) vimentin preincubation group. Nuclei were stained with DAPI (blue), and M. pneumoniae and rP1-C were stained with Cy3 (red). All signals were merged (yellow) (×400, in oil, LSCM; Zeiss).

**FIG 8:**
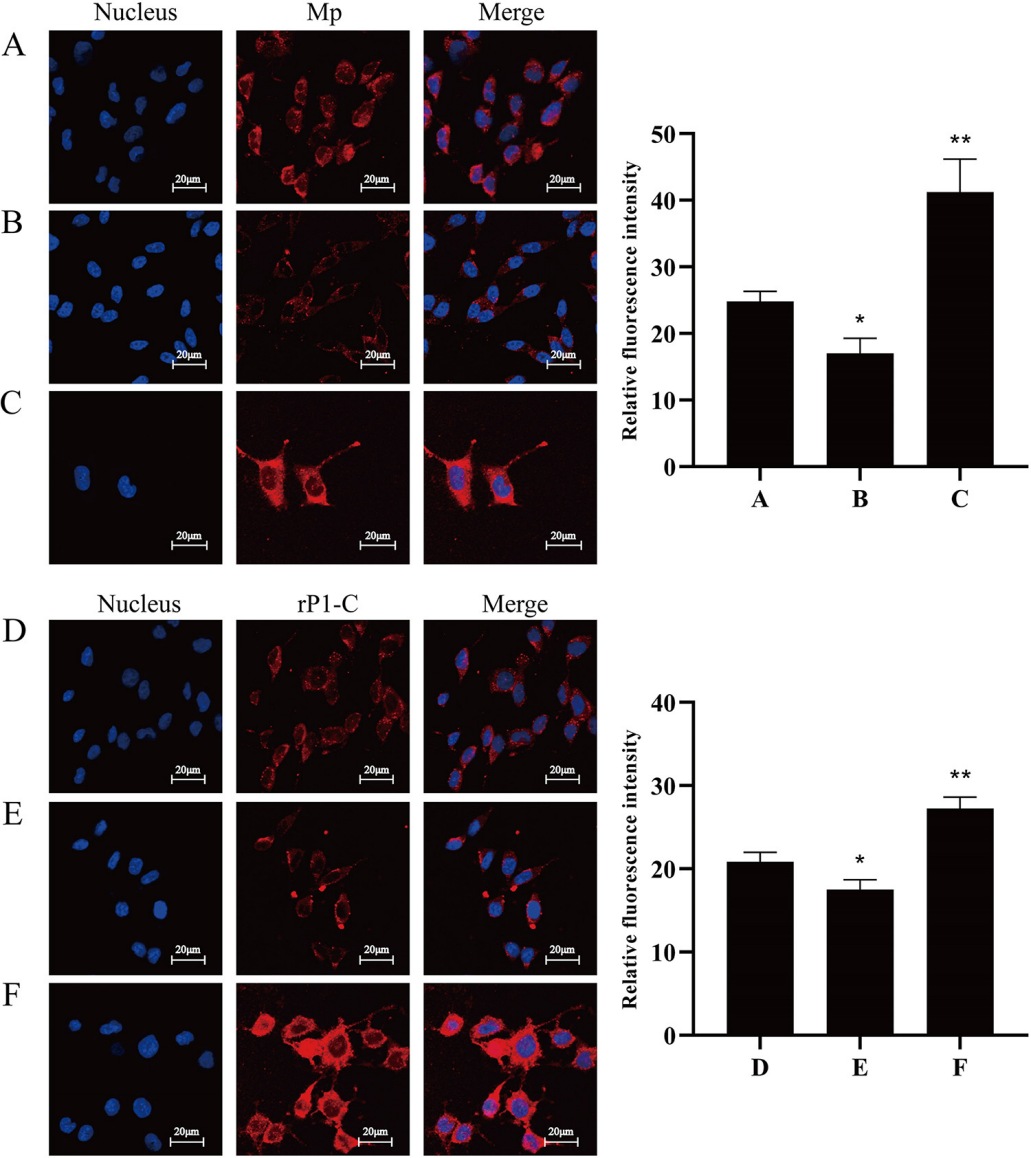
Effect of vimentin expression level on adhesion. (A and D) Control siRNA group; (B and E) vimentin siRNA group; (C and F) vimentin overexpression group. Nuclei were stained with DAPI (blue); M. pneumoniae and rP1-C were stained with Cy3 (red). All signals were merged (yellow) (×400, in oil, LSCM; Zeiss).

Adhesion and adhesion inhibition experiments were performed to test whether the binding of rP1-C and M. pneumoniae to BEAS-2B cells could be inhibited by vimentin or its antibody. M. pneumoniae adhered to the surface of the BEAS-2B cell membrane (Fig. 7A, red fluorescence). Still, pretreatment of the cells with anti-vimentin antibody partly inhibited the adhesion of M. pneumoniae (Fig. 7B). Similarly, after M. pneumoniae was treated with vimentin, the red fluorescence associated with the cells was decreased (Fig. 7C). These results corroborated the hypothesis that pretreatment with vimentin could inhibit the adhesion of M. pneumoniae to cells. Likewise, the adhesion of rP1-C to cells was similar to that of M. pneumoniae, wherein the red fluorescence was distributed on the surface of the cell membrane, indicating that rP1-C adhered to the cell membrane (Fig. 7D). Compared with that in Fig. 8D, the red fluorescence was significantly reduced after the P1 protein was preincubated with vimentin or the cells with anti-vimentin (Fig. 7E and F), proving that adhesion of rP1-C to BEAS-2B cells was inhibited by vimentin and its antibody. To recap, vimentin and its antibody could inhibit the adhesion of M. pneumoniae and rP1-C to BEAS-2B cells, indicating that vimentin was closely related to the adhesion of M. pneumoniae and rP1-C to BEAS-2B cells.

### Vimentin levels affect the adhesion of rP1-C and M. pneumoniae to BEAS-2B cells.

To confirm that vimentin was the target for adhesion of M. pneumoniae to cells more intuitively, indirect immunofluorescence was performed on BEAS-2B cells in which vimentin expression was decreased by the introduction of interfering vimentin small interfering RNA (siRNA). In contrast, in other BEAS-2B cells, vimentin was overexpressed. As shown in Fig. S1, vimentin expression in vimentin siRNA-transfected cells was significantly reduced, while vimentin expression was upregulated considerably for the vimentin-overexpressing cells. For the vimentin siRNA-transfected cells, the adhesion of rP1-C and M. pneumoniae to BEAS-2B cells was significantly lower than that of the control cells (Fig. 8A, B, D, and E). The vimentin-overexpressing BEAS-2B cells had a significantly higher level of adhesion of rP1-C and M. pneumoniae than the wild-type cells (Fig. 8C and F). These results verified that the expression level of vimentin may affect the adhesion of rP1-C and M. pneumoniae to BEAS-2B cells.

## DISCUSSION

M. pneumoniae was first isolated from the sputum specimen of a patient with atypical pneumonia and is the leading cause of community-acquired pneumonia (CAP) in children (21). Although pneumonia caused by M. pneumoniae is often self-limited, refractory pneumonia and extrapulmonary complications can occur in serious cases. The course of mycoplasma pneumonia is continuous and repeated, which brings great pain to patients and can result in long-term sequelae (22, 23). However, with the increasing severity of antibiotic abuse in recent years, macrolide-resistant M. pneumoniae strains have appeared in many regions (24, 25), resulting in hospitals losing treatment options for M. pneumoniae infections. Thus, recent studies on the pathogenic mechanism of M. pneumoniae have attempted to provide new strategies for the prevention and treatment of M. pneumoniae infections. Attachment to host respiratory epithelial cells is the initial step for the pathogenesis of M. pneumoniae. M. pneumoniae expresses a variety of adhesion proteins, such as P30, P40, and P90, which complement with P1 protein to form the tip structure of M. pneumoniae, so that it can firmly adhere to the surface of the cell membrane and absorb nutrients from the host cells. The survival of M. pneumoniae on the surface of host cells indicates that there must be some receptors that can bind to M. pneumoniae.

There have been a few studies on the receptors of M. pneumoniae. In 1988, Krivan et al. (26) reported that M. pneumoniae can attach to glycolipids containing terminal Gal(3SO_4_)/β1 residues. Subsequently, Roberts et al. (27) found that glycoproteins containing terminal NeuAcα2-3Galβ1-4GlcNAc sequences participated in the adhesion of M. pneumoniae. However, the interaction between receptors and ligands is not one to one. The same pathogen may use multiple receptors, and the same receptor may be involved in the infection process of various pathogens. Widjaja et al. found that the C-terminal region of P1 selectively binds to cytokeratin and vimentin, which are intermediate modifications of the A549 cytoskeleton (11). This indicates that a specific P1 protein may have various diverse interacting proteins and further confirms the reliability of our results. Previous studies indicated that M. pneumoniae might mediate adhesion by recognizing sialic acid oligosaccharides on the surface of host cells *in vitro* (27). Since the pivotal role of P1 in the adhesion of M. pneumoniae has been certified, it is increasingly conceded that this protein mediates the adhesion of M. pneumoniae to host cells through its interaction with sialic acid oligosaccharides. However, Vizarraga et al. revealed that M. pneumoniae binds to sialic acid through P40/P90 rather than P1 protein (28). We speculate that this phenomenon may be due to the fact that P1 protein can affect the formation of tip structure of *M. pneumoniae* or that P1 protein can also bind to other receptors on the host cell membrane. Notably, although P40/P90 contains sites for binding to sialic acid cell receptors, P1 is considered a primary determinant of adhesion and sliding motifs of M. pneumoniae (15). Therefore, P1 protein and its corresponding receptor are still worthy of further study. Svenstrup et al. showed that only the C-terminal region of P1 protein was exposed on the surface of M. pneumoniae and that the amino acids involved in cell adhesion were amino acids 1382 to 1394 in the C-terminal region (14). This is comparatively consistent with the observation of Drasbek et al. (13), that is, that P1 binds directly to the host receptor at amino acids 1347 to 1396 in the C-terminal region. Therefore, to study the adhesion characteristics of P1, we prepared a recombinant protein containing amino acids 1160 to 1498 in rP1-C (29). We successfully screened the membrane protein fraction of BEAS-2B bronchial epithelial cells for proteins specifically binding to rP1-C using a modified VOPBA method and LC-MS. The results showed that the two proteins with the highest scores were vimentin and β-4-tubulin. However, the results of far-Western blotting and coimmunoprecipitation validated that β-4-tubulin could not interact with rP1-C or that the binding between them was weak. Therefore, we did not conduct a follow-up experiment on tubulin. Subsequently, we confirmed that rP1-C and vimentin could interact with each other specifically, which contributed to the binding of rP1-C and M. pneumoniae to the surface of BEAS-2B cells. The adhesion and adhesion inhibition assays demonstrated that vimentin played a vital role in the adhesion process. Since there is more than one M. pneumoniae receptor on the cells, the adhesion of M. pneumoniae could not be blocked entirely. In further experiments, we found that the amounts of rP1-C and M. pneumoniae adhering to the cells corresponded to the expression level of vimentin. The results indicated that vimentin might be the primary M. pneumoniae receptor on BEAS-2B cells. Interestingly, in this study, a small amount of M. pneumoniae was also observed in the nuclear area of the infected cells under a confocal microscope. Pathogens can invade cells by binding to receptors, but whether M. pneumoniae can invade BEAS-2B cells by binding to vimentin remains to be determined.

Vimentin, a cytoskeletal protein, belongs to the family of type III intermediate filament proteins and is mainly expressed by mesenchymal cells of mesodermal origin, such as fibroblasts, endothelial cells, neutrophils, and macrophages (30). Vimentin not only plays a role in maintaining cell morphology, movement, and division (31) but also plays a role in pathogen infection (32), tumor invasion, and signal transduction (33). Vimentin is involved in cell adhesion and migration (34), and its absence can affect wound healing and tissue repair (35). In addition, vimentin is also considered a typical marker for the epithelial-to-mesenchymal transition (EMT) (36), and its abnormal expression in tumor cells can increase their invasiveness. For example, in colorectal cancer (37), lung cancer (38), breast cancer (39), and glioblastoma (40), increased expression of vimentin enhanced cell migration and invasion.

Multiple studies have already shown that in the pathogen infection process, vimentin can play an essential role both intracellularly and extracellularly. During the process of infection of Vero E6 cells by severe acute respiratory syndrome coronavirus (SARS-CoV), vimentin was involved in the virus’s entry into cells through direct interaction with the viral spike protein in a spike-ACE2 (angiotensin-converting enzyme 2) complex. With decreasing vimentin expression, the uptake of viral spike protein by cells also dramatically decreases (41). Human enterovirus 71 (EV71) interacts with vimentin as the adhesion receptor, facilitating transport across the blood-brain barrier and allowing the virus to enter the brain parenchyma (42, 43). Streptococcus agalactiae (group B streptococcus [GBS]) (44) and Listeria monocytogenes (45) were confirmed to penetrate the blood-brain barrier in the same way, causing life-threatening meningitis and encephalitis. Except for human papillomavirus 16 (HPV-16) (46), after most pathogens invade the host through vimentin, the expression of vimentin is induced to increase through various mechanisms. The upregulation of vimentin provides conditions for the adhesion and invasion of more of the pathogen, thereby ensuring more significant colonization. In this study, we confirmed that the decreased expression of vimentin reduced the adhesion of M. pneumoniae to host cells, while the increased expression of vimentin led to the increased adhesion of M. pneumoniae. However, whether the interaction between M. pneumoniae and vimentin can upregulate the expression of vimentin in host cells remains to be verified. For bluetongue virus, vimentin drives the egress of the mature virus by binding to the outer capsid protein VP2, and the disruption of vimentin can cause virus particles to be unable to release and accumulate in infected cells (47). In addition, vimentin is involved in synthesizing the viral replication complex of dengue virus (48, 49) and human immunodeficiency virus (50). It seems that vimentin also plays a positive role in the proliferation and release of pathogens in host cells. Therefore, we assume that the colonization and proliferation of M. pneumoniae in host cells depend on the same mechanism.

The signaling pathways involved in vimentin regulation are complex and variable. Vimentin can regulate the activity of Rac1 and promote the directed migration of cells by forming a complex with β4 integrin (51). It also promotes axonal growth through interaction with insulin-like growth factor 1 receptor (IGF1R) (52), activates dectin-1, and promotes the formation of atherosclerosis (53) or interacts with Jagged, adjusting the balance of Jagged and Dll4 signaling (54). Among them, the most important thing that has aroused our attention is that vimentin can act as the ligand for the NKp46 receptor of human NK cells to guide them to kill infected cells (55). We have to consider whether vimentin can play a role not only as an adhesion receptor but also as a ligand mediating the killing of other pathogens (including M. pneumoniae), which needs further investigation. In addition, the regulation of NLRP3 inflammasomes by vimentin indicated that vimentin could also play a significant role in the inflammatory response (56). After M. pneumoniae infects host cells, it could cause the upregulation of a variety of cytokines and chemokines, leading to the recruitment and infiltration of inflammatory cells. Therefore, the cell signal changes caused by the combination of the P1 protein of M. pneumoniae with vimentin need further study.

In conclusion, we demonstrated that the M. pneumoniae P1 protein can interact with vimentin and that the adhesion of M. pneumoniae and recombinant P1 protein to BEAS-2B cells was affected by the expression level of vimentin. Attachment could be blocked by pretreatment with vimentin antibody, indicating that vimentin might be one of the receptors for M. pneumoniae adherence to host cells. Gavitt et al. showed that monoclonal antibodies to P1 adhesion protein could interfere with the adhesion of Mycoplasma pneumoniae (57), and our study found that the change of vimentin expression and the blocking of vimentin would also affect the adhesion of rP1-C and Mycoplasma pneumoniae, which provided a new idea for the prevention and treatment of Mycoplasma pneumoniae infection.

## MATERIALS AND METHODS

### Cell culture and construction of a stable cell line overexpressing vimentin.

The human bronchial epithelial cell line previously utilized in our laboratory, BEAS-2B, was purchased from the ATCC (CRL-9609). Cells were cultured in 25-cm^2^ dishes (Corning, USA) with bronchial epithelial cell growth medium (BEGM; Lonza; cc-3170) and incubated in a 5% CO_2_ incubator at 37°C.

The lentivirus vector CMV-GFP-puro-vimentin was constructed by Genscript (Nanjing, China). The normal cell line was plated at 2 × 10^6^ cells per well in 6-well plates and incubated for 48 h. A fresh medium containing Polybrene with a final concentration of 8 μg/mL was added and incubated at 37°C for 30 min. Then the cells were infected with the lentivirus vector at multiplicities of infection (MOI) of 0, 1, 3, 5, 7, and 9 and cultured for 24 h. The virus-containing medium was discarded the next day and replaced with fresh medium for another 24 h. To screen for successfully infected cells, fresh puromycin medium was replaced at a final concentration of 4 μg/mL. After the cells were cultured in the medium containing puromycin for two generations, reverse transcription-quantitative PCR (RT-qPCR) and Western blotting were performed to determine vimentin expression.

### M. pneumoniae culture.

M. pneumoniae (ATCC 15531) was grown in plastic centrifuge tubes with PPLO broth (Difco, BD, USA) at 37°C until the late exponential phase, then aliquoted into 1-mL stocks, and stored at −20°C. Cells were harvested by centrifugation at 12,000 × *g* for 10 min, washed three times with phosphate-buffered saline (PBS; HyClone), and resuspended in BEGM.

### M. pneumoniae P1 protein fragment expression.

The recombinant protein was obtained from the DNA sequence of the *p1* gene of M. pneumoniae for expression and purification. The *p1* gene sequence corresponding to amino acid residues 1160 to 1498 of P1 adhesin was codon optimized, synthesized, and inserted between the EcoRI and XhoI sites in the pET-30a(+) vector (Genscript, Nanjing, China). The recombinant vector, rP1-C/pET-30a(+), was transformed into Escherichia coli (strain Rosetta) to express the hexahistidine-tagged protein. The cells carrying rP1-C/pET-30a(+) were cultured at 37°C and then induced by isopropyl β-d-thiogalactoside (IPTG; Solarbio) at a final concentration of 0.8 mM for 4 h. Cells were pelleted by centrifugation at 5,000 × *g* for 15 min, resuspended in lysis buffer (200 mM NaCl, 50 mM Tris, 1% Triton X-100, 20% glycerol [pH 7.8]), and sonicated on ice. The cell lysates were centrifuged at 12,000 × *g* for 30 min; the supernatants were collected and the presence of protein was detected by SDS-PAGE. The supernatants were loaded onto a column of Ni-nitrilotriacetic acid (NTA) beads (Qiagen, Germany). The column was washed with washing buffer (50 mM Tris, 300 mM NaCl, 10% glycerol) and eluted with a linear gradient of 5 to 250 mM imidazole. The purified protein was washed with PBS, concentrated on a regenerated cellulose membrane with a molecular weight cutoff of 10 kDa (Millipore, USA), and then stored at −80°C.

### Preparation of antibody.

Three female New Zealand rabbits (8 weeks old; 2.0 to 2.2 kg) were used. One of them was injected subcutaneously with PBS emulsified in Freund’s adjuvant (Sigma), and the remaining two were injected with 200 μg of purified rP1-C emulsified in Freund’s adjuvant, every 2 weeks (four times total). The blood was drawn, serum was centrifuged at 13,000 × *g*, and the supernatants were collected. The supernatants were purified as described as follows. The supernatants were mixed with equal volumes of PBS, and saturated ammonium sulfate solution was added dropwise and allowed to react for 1 h at 4°C. After centrifugation at 13,000 × *g*, the supernatants were discarded, and the residue was dissolved in PBS. A saturated ammonium sulfate solution was again added, and the dosage was gradually reduced. Lastly, the partially purified serum was collected and further purified by passage through a column of rP1-C-conjugated CNBr-activated Sepharose 4B (GE Healthcare, Sweden), as described by Zeng et al. (58). SDS-PAGE and Western blotting were performed to verify that the purification was successful.

### Western blotting and far-Western blotting.

The cell protein samples were extracted, mixed with sample loading buffer, boiled for 10 min, then subjected to SDS-PAGE, and transferred to a polyvinylidene difluoride (PVDF) membrane. The membrane was blocked with 5% skim milk in Tris-buffered saline containing 0.05% Tween 20 (TBST) at room temperature for 2 h and then incubated with the corresponding primary antibody at 37°C for 2 h. Subsequently, the membrane was incubated with horseradish peroxidase (HRP)-conjugated secondary antibody (1:10,000; Biosharp). Lastly, protein bands were visualized using an automated gel documentation and analysis system (Gene Company Limited, China).

For far-Western blotting, the rP1-C protein was mixed with sample loading buffer and subjected to SDS-PAGE as before, except that the PVDF membrane was first preincubated with cell membrane proteins (1 mg/mL) at 4°C overnight. All other steps were the same as for traditional Western blotting.

### Modified VOPBA and LC-MS.

A modified VOPBA was performed as described previously (59). Cells were washed twice with PBS, detached with trypsin (Gibco), and lysed in radioimmunoprecipitation assay (RIPA) lysis buffer (Millipore) containing phenylmethylsulfonyl fluoride (PMSF; Sigma). After sonication at 4°C for 10 min, the lysates were centrifuged at 2,500 × *g*, and the supernatants were collected and centrifuged at 16,000 × *g* for 30 min. Pellets were resuspended in PBS and then transferred to a PVDF membrane (Millipore) after SDS-PAGE treatment. The membrane was blocked with 5% skim milk in TBST at room temperature for 2 h and then incubated with rP1-C (1 mg/mL) overnight at 4°C. After washing three times with TBST, the membrane was incubated with the purified anti-rP1-C antibody (1:50) at 37°C for 2 h and then with HRP-conjugated goat anti-rabbit IgG antibody (1:10,000; Biosharp) at 37°C for 1 h. Protein bands were visualized using an automated gel documentation and analysis system (Gene Company Limited, China). After confirming the molecular weight of the band, the membrane protein suspension was subjected to SDS-PAGE, and the target strip was cut off for liquid chromatography-mass spectrometry (LC-MS) analysis, which was carried out by Guangzhou Huijun Biotechnology Co. Ltd.

### Distribution of target proteins on the cell membrane.

BEAS-2B cells were seeded into 24-well plates (Thermo Fisher, USA) and incubated overnight in 5% CO_2_ at 37°C. The cells were then washed with PBS, fixed with 4% paraformaldehyde, blocked with BEGM, and incubated with the anti-vimentin antibody (1:200; ab8069; Abcam, UK) or anti-tubulin antibody (1:200; OTI3C12; Invitrogen, USA) for 2 h at 37°C. Cells were washed and incubated with 150 μL of fluorescein isothiocyanate (FITC)-conjugated goat anti-mouse IgG (1:100; SA00003-1; Proteintech, USA) for 1 h at 37°C. DAPI (4,6-diamidine-2-phenylindole dihydrochloride; Beyotime, China) was added to visualize nuclear DNA. Images were captured with an inverted fluorescence microscope (Nikon, Japan) at a magnification of ×100. Images were collected sequentially to prevent cross talk between the fluorophores.

### Colocalization assay.

BEAS-2B cells cultured on coverslips were fixed with 4% paraformaldehyde and blocked with BEGM. Before incubation with primary antibody, the cells were preincubated with 150 μL of M. pneumoniae (1 × 10^7^ colour change unit (CCU)/mL) or rP1-C (1 mg/mL) for 2 h. For double immunofluorescence, cells were incubated with 200 μL of rabbit anti-vimentin antibody (1:200; cell signaling technology (CST), USA) or anti-tubulin antibody (1:200; Sigma, USA) and mouse anti-M. pneumoniae antibody (1:50; Invitrogen, USA) at 4°C overnight. Subsequently, the cells were incubated with 200 μL of FITC-conjugated goat anti-rabbit IgG and cyanine 3 (Cy3)-conjugated goat anti-mouse IgG (1:100; SA00009-1; Proteintech, USA) at 37°C for 1 h. The rP1-C-preincubated cells were incubated with 200 μL of mouse anti-vimentin antibody (1:200; Abcam) or anti-tubulin antibody (1:200; Invitrogen) and rabbit anti-rP1-c antibody at 4°C overnight. Subsequently, the cells were incubated with 200 μL of FITC-conjugated goat anti-mouse IgG and Cy3-conjugated goat anti-rabbit IgG (1:100; Proteintech) at 37°C for 1 h at room temperature. The remaining steps were the same as for the distribution assay. The treatment of the tubulin group was the same as described above. Images were captured with a confocal microscope (Zeiss, Germany) at a magnification of ×40. Images were collected sequentially to prevent cross talk between the fluorophores. Intensities of fluorescence staining were analyzed using ImageJ.

### Coimmunoprecipitation assay.

Protein A agarose beads were diluted with PBS to a 50% suspension and mixed with 50 μL of rP1-C antibody (1:20), anti-vimentin antibody (1:50), anti-tubulin antibody (1:50), or anti-IgG antibody (1:50; Servicebio, China) and incubated at 4°C overnight. After the beads were washed three times with PBS, 50 μL of rP1-C and cell membrane protein suspension were added, and the mixture was shaken on ice for 6 h. The excess unbound protein was washed away with PBS, and the remaining antigen-antibody complexes were resuspended with 40 μL of PBS. The proteins were visualized and identified by Western blotting.

### ELISA.

RP1-C was diluted to 40 μg/mL with coating buffer (15 mM Na_2_CO_3_, 35 mM NaHCO_3_ [pH 9.6]), and the diluted rP1-C was used to coat an ELISA plate (100 μL per well) at 4°C overnight. The plate was washed three times with phosphate-buffered saline with Tween 20 (PBST) and blocked with 5% skim milk in PBST at 37°C for 2 h. After washing, 50 μL of vimentin (0.2 mg/mL; ab73843; Abcam, UK) was added to each well and incubated at 37°C for 2 h. Anti-vimentin antibody (1:1,000; ab223871; Abcam) and secondary antibody (1:10,000; BS12478; Bioworld, China) were subsequently incubated in sequence, and the *A*_450_ was determined (Tecan; Infinite F50).

### Adhesion and adhesion inhibition assay.

To determine whether vimentin is a key receptor protein for the adhesion of M. pneumoniae and rP1-C to host cells, a series of adhesion and adhesion inhibition experiments were performed with the following groups: (i) blank control groups, (ii) anti-vimentin antibody preincubation group, and (iii) vimentin preincubation group. The cells were added to 24-well plates (5 × 10^5^ cells/well) and incubated at 37°C for 24 h. After cultivation for 48 h, group i was incubated with rP1-C (0.5 mg/mL) or M. pneumoniae (5 × 10^5^ CCU/mL) for 2 h. Group ii was preincubated with mouse anti-vimentin antibody (1:200) or rabbit anti-vimentin antibody (1:200) before incubation with rP1-C or M. pneumoniae, and group iii cells were incubated with rP1-C or M. pneumoniae that had been pretreated with vimentin (0.2 mg/mL). Nonimmune rabbit or rat IgG served as a control. Anti-rP1-C antibody and anti-M. pneumoniae antibody were incubated at 37°C for 2 h. Cy3-conjugated goat anti-rabbit IgG (1:200; A0516; Beyotime) or Cy3-conjugated goat anti-mouse IgG (1:200; A0521; Beyotime) was then added to the wells, followed by incubation for 1 h at 37°C. After staining with DAPI, the cells were imaged using a laser scanning confocal microscope (LSCM; Zeiss, Germany).

### Effect of vimentin expression level on adhesion.

To determine how vimentin expression affected the adhesion of M. pneumoniae and rP1-C to the cells, vimentin siRNA was introduced into BEAS-2B cells to reduce vimentin expression. At the same time, the lentivirus vector CMV-GFP-puro-vimentin was inserted for overexpression. For RNA interference, fresh medium was added to each well with 1.25 μL of siRNA (20 μM; SR322185; Origene, USA) and 1.25 μL of Lipofectamine 2000 and incubated at 37°C for 48 h. In another group, vimentin was overexpressed by the lentivirus vector CMV-GFP-puro-vimentin. After 48 h, each well was incubated with M. pneumoniae or rP1-C for 2 h. Nonimmune rabbit or rat IgG served as a control. After washing, the wells were incubated with primary antibody and secondary antibody in sequence to visualize the adherence of M. pneumoniae and rP1-C to BEAS-2B cells.

### Statistical analysis.

GraphPad Prism 7 was used to analyze all the data in this study. All the data are presented as means ± standard deviations (SD), and statistical analyses were performed by Student’s *t* tests. A *P* value of <0.05 was considered statistically significant.

### Ethics statement.

The animal protocol for this study was approved by the Animal. Welfare Committee of the University of South China and conducted by the institution’s regulations, and all efforts were made to minimize the animal’s suffering. This article does not contain any studies with human participants performed by any of the authors.

### Data availability.

All data included in this study are available upon request by contact with the corresponding author.
