# Supplementary material for: Vimentin Is an Attachment Receptor for Mycoplasma pneumoniae P1 Protein
Source: Microbiol Spectr. 2023 Mar 13;11(2):e04489-22. doi: 10.1128/spectrum.04489-22 (PMC10100666; doi:10.1128/spectrum.04489-22)
Supplement: Supplemental file 1 — Supplemental material. Download spectrum.04489-22-s0001.pdf, PDF file, 0.2 MB [file spectrum.04489-22-s0001.pdf]

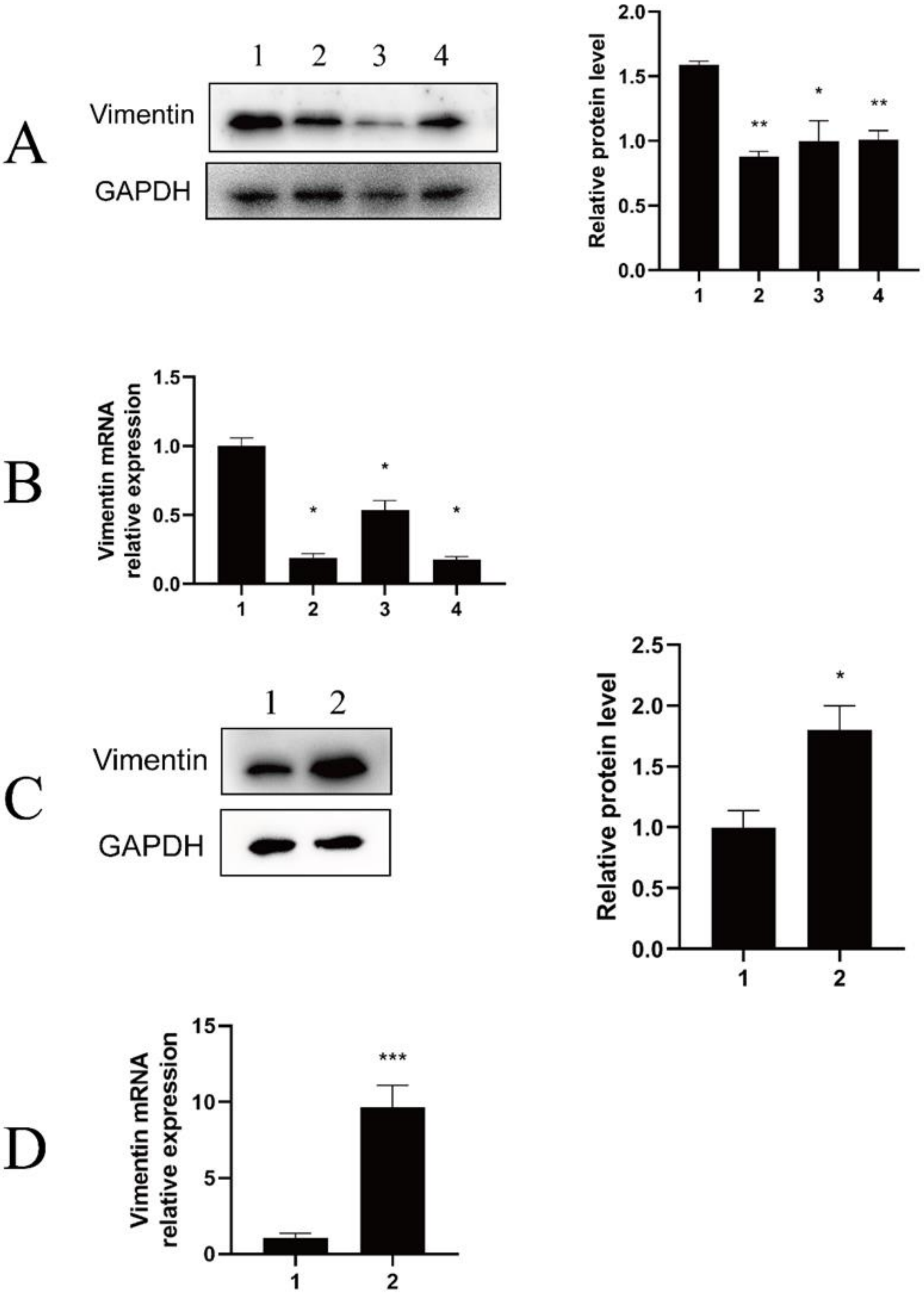

Supplemental 1. Expression of vimentin after transfection of siRNA or infection with lentivirus.

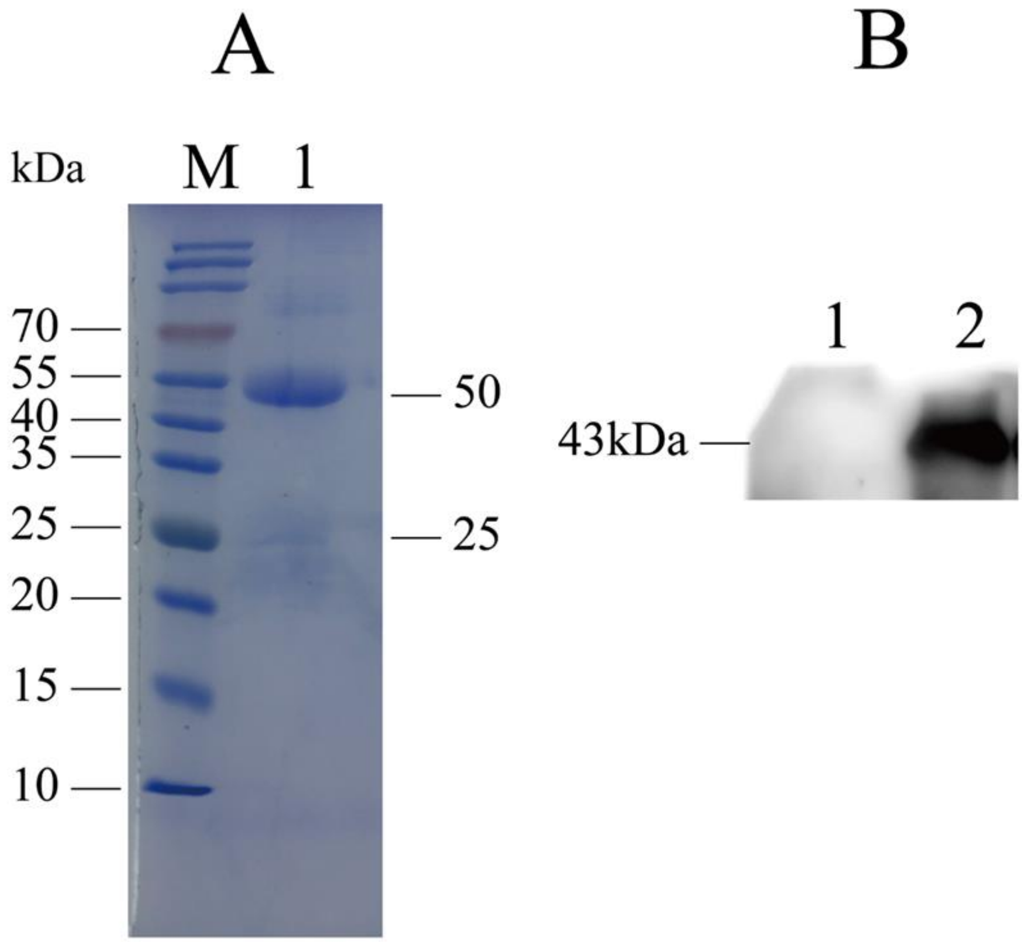

Supplemental 2. Purification and identification of anti-rP1-C antibody. (A) SDS-PAGE analysis of purified anti-rP1-C antibody. (B) Western blotting to detect antibody specificity. 1: empty E. coli suspension; 2: purified rP1-C (PVDF membrane was incubated with purified anti-rP1-C antibody).

Table S1. siRNA duplexes sequences

Name	Sequences
SR322185A	rCrArCrGrArUrGrArCrCrUrUrGrArArUrArArArArArUrUGC
SR322185B	rArArArArArUrCrUrUrGrUrGrCrUrArGrArArUrArCrUrUTT
SR322185C	rCrUrUrCrUrGrArUrUrArArGrArCrGrGrUrUrGrArArArCTA

Table S2. Primers sequences

Gene name	Forward (5′-3′)	Reverse (5′-3′)
VIM	TCGTGAATACCAAGACCTGCTCAATG	AATCCTGCTCTCCTCGCCTTCC
β-actin	ATCGTGCGTGACATTAAGGAGAAG	AGGAAGGAAGGCTGGAAGAGTG
